# Influence of Host Gender on Infection Rate, Density and Distribution of the Parasitic Fungus, *Hesperomyces virescens,* on the Multicolored Asian Lady Beetle, *Harmonia axyridis*

**DOI:** 10.1673/031.006.4201

**Published:** 2006-11-27

**Authors:** E. W. Riddick

**Affiliations:** ^1^ USDA-ARS, Biological Control of Pests Research Unit, 59 Lee Road, Stoneville, MS 38776, USA

**Keywords:** behavior, horizontal transmission, host-parasite interaction, predator

## Abstract

*Hesperomyces virescens* Thaxter (Laboulbeniales: Laboulbeniaceae) is a parasitic fungus that infects lady beetles (Coleoptera: Coccinellidae) via horizontal transmission between adults at overwintering and feeding sites. The differential behavior of male and female hosts could have profound effects on intensity of infection and positioning of fungus on the host's integument. The influence of host gender on infection rate, density and distribution of this parasite on the multicolored Asian lady beetle, *Harmonia axyridis* (Pallas) (Coleoptera: Coccinellidae), was determined at a feeding site. Adult *H. axyridis* were sampled from pecan, *Carya illinoinensis* (Wangenh.) K. Koch, trees in northern Mississippi, USA, during summer and early fall 2003–2004. Results indicated that the behavior of male or female beetles on pecan trees had only a limited effect on the intensity of infection. When averaged over the entire season, the percentage of *H. axyridis* infected with *H. virescens* was not influenced by host gender. In 2003, a seasonal average of 54 and 39% of males and females, respectively, were infected; whereas in 2004, 36 and 41% of male and female beetles, respectively, were infected. The percentage of males infected with *H. virescens* was correlated with the number of males captured at the site in 2003; infection rate decreased as male abundance increased. Infection rate did not correlate with female abundance in 2003 or male or female abundance in 2004. Host gender had a considerable effect on the density and distribution of the fungus. *Hesperomyces virescens* mature thalli were denser on male rather than female beetles. Also, thallus density was often greatest on the elytra, meso- and metathorax, and abdomen of males and elytra of females, than on other body parts, in 2003. In 2003 and 2004, approximately 59 and 97% and 67 and 96% of males and females, respectively, had mature thalli distributed on the elytra. Prevalence of *H. virescens* thalli on the dorsum of *H. axyridis* females suggests that mating behavior is important in fungal transmission. However, prevalence of thalli on the dorsum of *H. axyridis* males suggests that behaviors other than mating contribute to the transmission of *H. virescens* onto male beetles. Spread of *H. virescens* ascospores from infected to uninfected *H. axyridis* adults of different generations, at feeding sites, might be vital to maintaining stable populations of the fungus.

## Introduction

Ascomycetous fungi in the order Laboulbeniales are known as ectoparasites of millipedes, mites, and insects (Thaxter 1931; Tavares 1979, 1985; Tanada and Kaya 1993). The order contains nearly 2,000 described species worldwide, 80% of which parasitize beetles (Santamaria 2001; Weir and Blackwell 2004). As far as is known, fungi in this order are biotrophic; they survive only on the cells or tissues of living hosts (Richards and Smith 1954; Whisler 1968). Horizontal transmission of Laboulbeniales between adult hosts is the typical mode of infection. Vertical transmission is reported for several species that attack insects (such as termites, earwigs, and cockroaches) in which juvenile and adult stages coexist (Thaxter 1896; Whitney 1982). Infection occurs when the basal cell of a two-celled ascospore attaches to the cuticle of a susceptible arthropod. In Laboulbeniales species a haustorium is produced from the basal cell that penetrates through the cuticle extending into the hemocoel or into various tissues of the host (Tavares 1979; Weir and Beakes 1996). The cell of the ascospore undergoes repeated mitotic divisions to produce distinct parts of the determinate thallus. The thallus is composed of an array of cells that form the reproductive organs of the fungus (Tavares 1979). In some species, thalli reach maturity within 3 wk (Strandberg and Tucker 1974; De Kesel 1993) and can have a lifespan of up to 10 wk during summer conditions (De Kesel 1993).

Although Laboulbeniales species are generally considered to be rather avirulent and have little detrimental effect on hosts (Tavares 1979; Weir and Beakes 1995), there are some exceptions. Some species may reduce host life span and egg production (Strandberg and Tucker 1974), reduce mobility (Gemeno et al. 2004), or cause premature mortality when clusters of thalli on mouthparts and antennae impede feeding behavior (Bro Larsen 1952). *Hesperomyces virescens* Thaxter (Laboulbeniales: Laboulbeniaceae) is a parasitic fungus that infects lady beetles via horizontal transmission between adults at overwintering and feeding sites. Kamburov et al. (1967) reported that *H. virescens* infected up to 95% of the adults of a coccinellid, *Chilocorus bipustulatus* L., in citrus groves in Israel, resulting in premature mortality of hosts. However, others have suggested that lack of prey in the groves rather than infection by *H. virescens* was primarily responsible for the decline of *C.* *bipustulatus* populations (Applebaum et al. 1971).

Infection rate can be influenced by host density (or abundance); *Laboulbenia phaeoxanthae* infection rate was highest when the abundance of its carabid host was lowest and *vice versa* in the field (Zerm and Adis 2004). Others have suggested the opposite pattern; that is, infection rate was highest when host density was high rather than low (De Kesel 1993).

Host gender may influence the incidence of infection. Certain species have been known to attack only males or females (Benjamin and Shanor 1952) and male, rather than females, were more often infected by Laboulbeniales in the laboratory (Richards and Smith 1955; Riddick and Schaefer 2005). Infection can be restricted to specific positions on the host (Thaxter 1896; Richards 1952; Benjamin and Shanor 1952; Richards and Smith 1955; Benjamin 1965; Rossi and Kotrba 2004; Terada 2005). Thalli were more prevalent on the ventrum of males and the dorsum of female beetles (Benjamin and Shanor 1952; Whisler 1968; Andersen and Skorping 1991; Welch et al. 2001) and flies (Whisler 1968; Hedstrom 1994). *Laboulbeniales clivinalis* thallus density was greatest during the mating season of its carabid host, *Clivina fossor* (De Kesel 1995). Thallus distribution was affected by host gender; but only during the mating season of *C. fossor* (De Kesel 1995).

Physical contact between host males and females while mating is a primary route of horizontal transmission of Laboulbeniales (Benjamin and Shanor 1952; Whisler 1968; Welch et al. 2001). Distribution of *H. virescens* on the ventrum of males and dorsum of females of the two-spot ladybird beetle (*Adalia bipunctata*) corresponded to the position of a male on top of a female when *in-copula* in spring and summer of 1998 and 1999 in London (Welch et al. 2001). Garcés and Williams (2004) indicated that *H. virescens* infection was concentrated on the ventroposterior of *Harmonia axyridis* males and the dorsoposterior of females found in crop fields in Ohio in summer 2002.

Non-mating physical contact while in overwintering aggregations can also be important in the spread of fungus (De Kesel 1995). Riddick and Schaefer (2005) reported that *H. virescens* thalli were concentrated on the dorsum and ventrum of *H. axyridis* males within an overwintering aggregation in Pennsylvania, USA. They speculated that male beetles were more active at the overwintering site and often crawled on and over other conspecifics, thus promulgating the spread of fungus to both dorsal and ventral body surfaces, regardless of gender. In contrast, thalli were distributed primarily on the dorsum of *H. axyridis* females (Riddick and Schaefer 2005), as expected via host mating behavior. Since mating can occur at aggregation sites before beetles disperse in early spring (see Nalepa et al. 1996), both mating and non-mating contacts between infected and uninfected conspecifics are likely responsible for spread of this fungus.

The density and distribution of *H. virescens* on *H. axyridis* during the summer in North America might closely resemble what would be expected if host mating was primarily responsible for transmission, since *H. axyridis* adults are not known to undergo aestivation (i. e., facultative dormancy or reproductive diapause). Thus, the objectives of this study were to determine the influence of host gender on infection rate, and the density and distribution of *H. virescens* on *H. axyridis* during summer and early fall.

## Materials and Methods

### Collection of lady beetles

Lady beetles were collected from the foliage of four adjacent pecan trees (*Carya illinoinensis* (Wangenh.) K. Koch, growing on the campus of Mississippi State University, Oktibbeha Co., MS (117 m elevation, 33° 27.173 N, 88° 48.322 W). The four trees were within an area of approximately 0.24 ha; the average distance between trees was 22.8 m and tree height did not exceed 20 m in the summer of 2003. Beetles were captured from tree foliage, from July to October 2003 and June to October 2004, by extending an aerial insect net (38 cm diam, BioQuip www.BioQuip.com) over the outermost 10 to 20 cm of low-lying limbs of each tree. These accessible tree limbs usually hung from 2 to 7 m above the ground. Beetles were trapped within a 50-ml polyethylene vial (Daigger, www.daigger.com) and removed from the net. Usually 2, but sometimes up to 4, beetles were placed in the same vial. All vials were placed in a small cooler and returned to the laboratory.

Sampling was conducted between 0800 and 1300 hrs in 2003; 0900 and 1240 hrs in 2004. The mean ± SEM sample time per collection date was 47.7 ± 3.4 min and 49.3 ± 3.1 min in 2003 and 2004 seasons, respectively (n = 15 dates in 2003 and 2004 seasons). The mean ± SEM temperature and humidity (as recorded with a hand-held hygrometer/temperature recorder) at the end of each collection time period were 29.85 ± 0.8 °C and 59.1 ± 3.5 % RH in 2003 (n = 15 dates) and 29.4 ± 0.8 °C and 58.5 ± 3.5 *%* RH in 2004 (n = 15 dates). A total of 301 and 135 *H. axyridis* males and females, respectively, were captured during sampling periods in 2003. A total of 206 and 225 males and females, respectively, were captured in 2004.

### Infection rate

**Figure 1.**
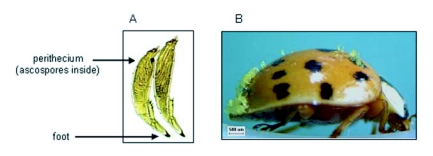
Image of mature thalli of the parasitic fungus *H. virescens* at 200X magnification (**A**) and an image of an infected *H. axyridis* male at 20X (**B**). The beetle was collected from a pecan tree, *Carya illinoinensis* (Wangenh.) K. Koch, in northern Mississippi (Washington Co.; 45.4 m elevation; 33° 25.204 N, 90° 54.913 W) on 26 June 2005 and cold-stored (at ∼ 10 °C) for 8 days before this image was produced. The age of this beetle was not known.

In 2003, beetles were sexed by noting the shape and appearance of the last two abdominal sternites of *H. axyridis* females and males (unpublished observations), and then placed, individually, in a clean 50-ml polyethylene vial and maintained in a refrigerator (at 3–5 °C). Often on the same day of collection (and no more than 3 days post-collection), beetles were removed from vials and examined for the presence of *H. virescens* mature thalli. Infected beetles (i. e., those individuals that had at least one mature thallus on the integument) were immediately preserved, individually, by dropping into a 1.5 ml polypropylene cryo-vial containing 70% ethanol (v/v). This was a very conservative estimate of infection. Morphological characters of mature thalli (rather than ascospores or developing thalli) are most often used to recognize Laboulbeniales species. The morphology of immature and mature *H. virescens* has been reported previously (see Weir and Beakes 1996). A mature thalli and an image of an infected lady beetle are shown in Figure 1. The percentage on *H. axyridis* males and females infected with *H. virescens* was determined for each collection date and for the season 2003 and 2004.

### Thallus density and distribution

The number of *H. virescens* mature, fully-developed, thalli on infected *H. axyridis* adults, in relation to host gender and body part (i.e., head, antennae, pronotum, etc.), was determined by using beetles captured from the field site in 2003. Data were subdivided by month of collection to determine if there was any trend in pattern of thallus density on beetles as the season progressed from July through October. Also, data were pooled across all collection dates to generate a seasonal average. Each beetle served as a replicate. Their age was unknown. A stereo-zoom microscope (10 X to 90 X magnification) was used for counting fungal thalli on body parts of males and females. Body parts (with code) were the following: antennae (An), head (He, including mouthparts, eyes, palpi), pronotum (Pro), left elytron (El-L), right elytron (El-R), prothorax (Pt), meso- and metathorax combined (MMt), abdomen (Ab), right foreleg (1 R), left foreleg (1 L), right midleg (2 R), left midleg (2 L), right hindleg (3 R), and left hindleg (3 L).

The percentage of infected beetles, (males vs females) with thalli distributed on key body parts, including elytra (left and right elytron combined), meso- and metathorax, and abdomen, was determined for each month from July through October, and for the entire season using the same 2003 data set. Percentage data were not all inclusive because the same individuals were used for the analysis of the three body parts on a given collection date. A total of 136 and 45 infected males and females, respectively, from a total of 28 collection dates, were collected in 2003.

In 2004, beetles were not preserved in alcohol; instead they were sexed and kept within individual 50-ml polyethylene vials and maintained in the refrigerator (at 3–5 °C). Usually on the same day of collection, beetles were examined for the presence of *H. virescens.* As before infected beetles were those that had one or more mature thallus on their integument. The laborious task of counting mature thalli on infected beetles was not undertaken in 2004. However, the percentage of infected beetles (males vs females) with thalli distributed on key body parts (as identified in 2003 counts) was determined for each month, June through October, and for the entire season in 2004. As before, percentage data were not all inclusive because the same individuals were used for the analysis of the three body parts on a given collection date. A total of 63 and 67 infected males and females, respectively, from a total of 27 collection dates, collected in 2004.

Voucher specimens of the fungus on *H. axyridis* adults are currently held at the USDA-ARS, Biological Control of Pests Research Unit, Stoneville, MS. The fungus was identified to species by A. Weir (SUNY, Syracuse, New York) for a previous study (Riddick and Schaefer 2005).

### Data analysis

Data were analyzed following a completely randomized design. A Pearson's correlation analysis was used to determine if the number of beetles captured on each date correlated with daily infection rate and a Student's *t*-test was used to determine the significance of host gender on season-long infection rate in 2003 and 2004. A two-factor analysis of variance (ANOVA) was used to test for the influence of host gender and body part on total number of mature *H. virescens* thalli per beetle for each month and for a pooled seasonal average in 2003. Three independent *t*-tests were used to determine the significance of host gender on the percentage of beetles harboring mature thalli on elytra, meso- and metathorax, and abdomen for monthly averages (except in June 2004, which had only a single collection date) and for a pooled seasonal average in 2003 and 2004. Absolute data were square-root transformed and percentage data were arcsine-transformed prior to analysis (Zar 1999) and a Holm-Sidak multiple comparison test was used for separation of means after ANOVA (SigmaStat 2004). Means were considered significantly different when *P* ≤ 0.05. Statistical analyses were performed with SigmaStat (2004) software. Only untransformed data are presented.

## Results

### Infection rate

**Figure 2.**
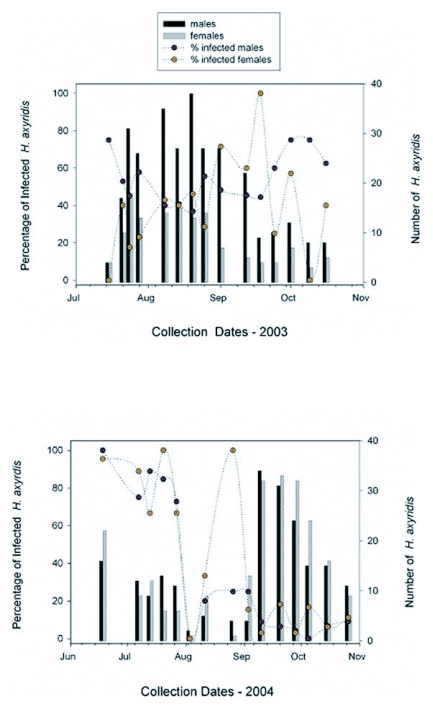
Percentage of *H. axyridis* adults infected with *H. virescens* and number of adults captured on pecan trees at an urban site during summer and fall 2003 (**A**) and 2004 (**B**). For percentage data, n = 15 observations per gender for both seasons. Circles and vertical bars illustrate percentage and absolute data, respectively. Collection dates included 15, 21, 24, 28 July; 8, 14 20, 25 August; 1, 12 18, 24 September; and 1, 9, 16 October 2003. In 2004 18 June; 7, 13 20, 27 July; 3, 11, 26 August; 3, 10 20, 28 September; and 5, 15, 26 October 2004.

The percentage of *H. axyridis* males infected with *H. virescens* never fell below 35% on any collection date in 2003 (Figure 2A). Infection rates for females fluctuated considerably; none were infected on one date in July and in October and 100% were infected on one date in September 2003. In 2003, a significant correlation between the percentage of males infected and the number of males captured was evident (*r* = -0.77; *P* < 0.001; n = 15 collection dates); infection rate increased as male abundance decreased. No correlation was found between the percentage of females infected and the number of females captured (*r* = 0.007; *P* = 0.98; n = 15). The mean ± SEM season-long infection rate in 2003 was 54.3 ± 34 and 39.4 ± 6.8% for males and females, respectively (n = 301 males, 135 females). Host gender had no influence on season-long infection rate in 2003 (*t* = 1.66 (28), *P* = 0.11; n = 30 observations).

In 2004, the percentage of *H. axyridis* males and females infected with *H. virescens* exceeded 65% from mid-June through July (Figure 2B). Less than 10 and 20% of males and females, respectively, were infected with *H. virescens* from mid-September through October 2004. The percentage of males or females infected with *H. virescens* was not correlated with the number of males or females captured (males: *r* = -0.05; *P* = 0.85; n = 15; females: *r* = -0.35; *P* = 0.20; n = 15). The mean ± SEM season-long infection rate in 2004 was 35.6 ± 9.8 and 40.8 ± 10.0% for males and females, respectively (n = 206 males, 225 females). Host gender had no influence on season-long infection rate in 2004 (*t* = 0.49 (28); *P* = 0.63, n = 30 observations).

Several other coccinellids were collected on pecan in this study. A single *Hippodamia convergens* Guerin-Meneville adult was captured on 3 September 2004 at the site; it was not infected. One *Olla v-nigrum.* (Mulsant) adult was captured at the site on 20 August 2003 and another adult was captured on 11 August 2004; neither one was infected with fungus. Two *Cycloneda sanguinea* (L.) adults were collected at the site in 2003 (one on 24 July and another on 16 October). Neither one was infected with *H. virescens.* The gender of these individuals was not determined.

### Thallus density and distribution in 2003

**Figure 3.**
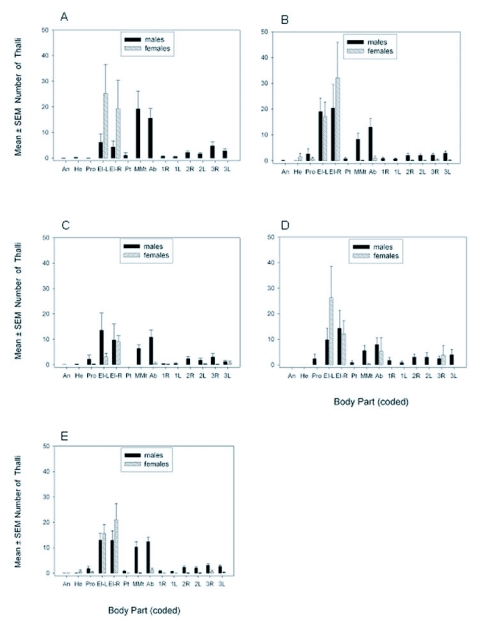
Mean ± SEM number *of H. virescens* mature thalli found on body parts of infected *H. axyridis* adults in July (**A**), August (**B**), September (**C**), October (**D**), and pooled for the season 2003 (**E**). Body parts (with codes) included antennae (An), head (He), pronotum (Pro), left elytron (El-L), right elytron (El-R), prothorax (Pt), mesoand metathorax (MMt), abdomen (Ab), right foreleg (1 R), left foreleg (1 L), right midleg (2 R), left midleg (2 L), right hindleg (3 R), and left hindleg (3 L). In July, n = 616 body part observations (37 males, 7 females); in August, n = 994 observations (51 males 20 females); in September, n = 602 observations (30 males, 13 females); in October, 406 observations (23 males, 6 females). For the season, n = 2, 618 observations (141 males, 46 females).

**Figure 4.**
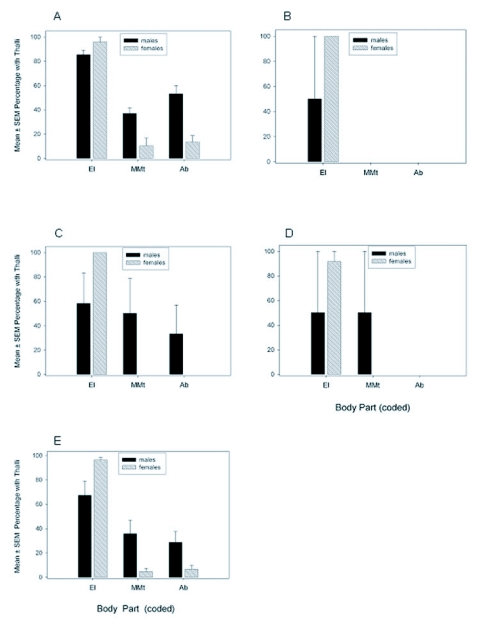
Mean ± SEM percentage of infected *H. axyridis* adults with *H. virescens* thalli on body parts in July (**A**), August (**B**), September (**C**), October (**D**), and pooled for the season 2003 (**E**). Body parts included elytra (El), meso- and metathorax (MMt), and abdomen (Ab). In July, n = 7 observations per body part (36 males, 7 females); in August, n = 8 observations (51 males 20 females); in September, n = 8 observations (30 males, 13 females); and October, n = 5 observations (19 males, 5 females). For the season 2003, n = 28 observations (136 males, 45 females).

In July 2003, the interaction of host gender and body part was significant (*F* = 6.7 (13, 588); *P* < 0.001). More *H. virescens* mature thalli were found on the elytra of *H. axyridis* females than males and more thalli were found on the mesoand metathorax, abdomen, midlegs and hindlegs of males than females (Figure 3A). Irrespective of body part, male rather than female beetles hosted more fungal thalli (*F* = 4.1 (1, 588); *P* = 0.04). Male beetles had more thalli on the ventrum than the dorsum; female beetles had thalli restricted to the dorsum. Irrespective of gender, more thalli were found on the elytra, abdomen and meso- and metathorax than other body parts (*F* = 7.6 (13, 588); *P* < 0.001). In July, 42 and 100% of infected *H. axyridis* males and females, respectively, had *H. virescens* mature thalli on the elytra (Figure 4A) and host gender was influential (*t* = 8.05 (5); *P* < 0.001). Only male beetles had mature thalli on the meso- and metathorax and abdomen during this month.

In August, the interaction of host gender and body part was significant (*F* = 3.0 (13, 966), *P* < 0.001). More mature thalli were found on meso- and metathorax and legs of males than females (Figure 36). Host gender had a significant influence on thallus density (*F* = 10.1 (1, 966); *P* = 0.002); males hosted more thalli than females. Thalli were found on the dorsum and ventrum of males, but females had thalli primarily on the dorsum. Body part had a significant influence on thallus density (*F* = 19.1 (13, 966); *P* < 0.001), with more thalli present on the elytra than other body parts. In August, more than 75% of infected males and females had mature thalli on the elytra (Figure 46) and host gender did not have a significant influence on thallus distribution (*t* = 2.2 (6); *P* = 0.07). A greater percentage of males than females had mature thalli on the meso- and metathorax (*t* = 6.3 (6); *P* < 0.001) and abdomen (*t* = 2.9 (6); *P* = 0.03).

In September, *H. virescens* thalli appeared somewhat less dense on the dorsum of females than in any other month (Figure 3C). The interaction of host gender and body part was significant (*F* = 2.7 (13, 574); *P* = 0.001); more mature thalli were found on meso- and metathorax and abdomen of males than females. Host gender had a significant influence on thallus density (*F* = 13.5 (1, 574); *P* < 0.001); males hosted more thalli than females. Male beetles had thalli situated on the dorsum and ventrum, whereas, females had thalli primarily on the dorsum. Regardless of gender, body part had a significant influence on thallus density (*F* = 6.8 (13, 574); *P* < 0.001); more thalli were present on the elytra than other body parts, except the mesoand metathorax and abdomen. In September, 55 and 100% of males and females, respectively, had mature thalli on elytra (Fig. 4C); host gender was influential (*t* = 12.6 (6); *P* < 0.001). Only male beetles had mature thalli on the meso- and metathorax during this month. A greater percentage of males than females had mature thalli on the abdomen (*t* = 3.7 (6); *P* = 0.01).

In October, the interaction of host gender and body part was not significant (*F* = 1.5 (13, 378); *P =* 0.11; Figure 3D). Host gender had no significant influence on thallus density (*F* = 1.7 (1, 378); *P* = 0.20). Regardless of host gender, body part had a significant influence on thallus density (*F* = 7.0 (13, 378); *P* < 0.001); more thalli were present on the elytra than other body parts, except the abdomen. In October, 62 and 100% of males and females, respectively, had mature thalli on the elytra (Fig. 4D) and host gender was influential (*t* = 21.3 (3); *P* < 0.001). Host gender had no significant influence on thallus distribution on the meso- and metathorax (*t* = 1.8 (3); *P* = 0.16) or abdomen (*t* = 2.1 (3); *P* = 0.12), because of low sample size.

When the density data were pooled across the season, July-October 2003, the interaction of host gender and body part was significant (*F* = 11.2 (13, 2590); *P* < 0.001). More thalli were found on the left and right elytron of females than males, and more thalli were found on the meso- and metathorax, midlegs and hindlegs of males than females (Figure 3E). Females averaged more than 15 mature thalli on both the left and right elytron in 2003 and males averaged more than 10 mature thalli on the meso- and metathorax as well as on the abdomen. Significantly more mature thalli were found on males than females, irrespective of body part, for the season (*F* = 28.1 (1, 2590); *P* < 0.001). Significantly more thalli were present on the elytra than on any other body part (*F* = 37.1 (13, 2590); *P* < 0.001). Relatively few adults had thalli on antennae or mouthparts and thallus density on these body parts was very low.

When all distribution data were averaged across the 2003 season, approximately 59 and 97% of infected *H. axyridis* males and females, respectively, had mature thalli on elytra (Figure 4E). A greater percentage of females than males had mature thalli on the elytra (*t* = 9.9 (26); *P* < 0.001) and a greater percentage of males than females had mature thalli on the meso- and metathorax (*t* = 7.6 (26); *P* < 0.001) and abdomen (*t* = 9.3 (26); *P* < 0.001).

### Thallus distribution in 2004

**Figure 5:**
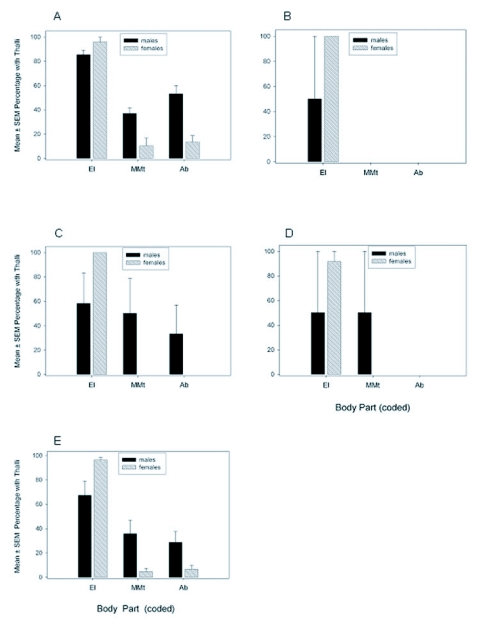
Mean ± SEM percentage of infected *H. axyridis* adults with *H. virescens* thalli on body parts in July (**A**), August (**B**), September (**C**), October (**D**), and pooled for the season 2004 (**E**). Body parts included elytra (El), meso- and metathorax (MMt), and abdomen (Ab). In June, n = 2 observations per body part (including 16 males, 21 females); in July, n = 8 observations (36 males, 26 females); in August, n = 4 observations (2 males, 4 females); in September, n = 6 observations (7 males, 10 females); and October, n = 4 observations (2 males, 6 females). For the season 2004, n = 27 observations (63 males, 67 females).

In June 2004 (only one collection date), more than 90% of infected *H. axyridis* males and females had mature *H. virescens* thalli on the elytra and a considerably lower percentage had thalli on meso- and metathorax and abdomen. In July, 86 and 96% of *H. axyridis* males and females, respectively, had mature thalli on the elytra (Figure 5A) and host gender was marginally influential (*t* = 2.4 (6); *P* = 0.05). A greater percentage of males than females had thalli on the meso- and metathorax (*t* = 2.8 (6); *P* = 0.03) and abdomen (*t* = 3.7 (6); *P* = 0.01) during this month. In August, at least 50 and 100% of infected males and females, respectively, had mature thalli on the elytra (Figure 5B). Host gender was not influential (*t* = 1.0 (2); *P* = 0.4), because of high variance. Neither males nor females had *H. virescens* mature thalli on the meso- and metathorax or abdomen. In September, 58 and 100% of infected males and females, respectively, had mature thalli on the elytra (Fig. 5C). Host gender was not influential (*t* = 1.6 (6); *P* = 0.15), because of high variance. Only male beetles had mature thalli on the meso- and metathorax and abdomen during this month. In October, 50 and 92% of infected males and females, respectively, had mature thalli on the elytra (Figure 50) and host gender was not influential (*t* = 0.97 (3); *P* = 0.40), because of high variance and low sample size. Only males had mature thalli on the meso- and metathorax; neither males nor females had thalli on the abdomen.

When all thallus distribution data were pooled across the 2004 season, approximately 67 and 96% of infected *H. axyridis* males and females, respectively, had mature thalli on the elytra (Figure 5E). A greater percentage of females than males had mature thalli on the elytra (*t* = 2.6 (25); *P* = 0.02) and a greater percentage of males than females had mature thalli on the meso- and metathorax (*t* = 2.8 (25); *P* = 0.01) and abdomen (*t* = 2.1 (25); *P* = 0.045).

## Discussion

### Infection rate

Although this study only estimated *H. virescens* infection of *H. axyridis* at one site, the fact that seasonal infection rates were (on average) above 35% in consecutive seasons suggests that this parasite-host association is established in northeastern Mississippi. In addition, *H. virescens* infection rates ranging from 20 to 49% (n = 547) and 8 to 32% (n = 656) of *H. axyridis* males and females, respectively, were observed at man-made structures in northeastern and southwestern MS during early to mid-November 2003 (unpublished observations). *Harmonia axyridis* was first established in North America in Louisiana and Mississippi in 1988 and 1990, respectively (Chapin and Brou 1991), and has become an important predator of aphids in several agroecosystems (see Brown 2004; Rutledge et al. 2004), as well as a nuisance pest in houses (see Nalepa et al. 1996; Riddick and Aldrich 2004). In any case, *H. axyridis* has not been reported previously to serve as host of *H. virescens* in the southeastern USA, until now.

*H. virescens* was found on *H. convergens* in Alabama (Thaxter 1931), on O. *v-nigrum* in Fiji (Weir and Beakes 1996) and Georgia, USA (T. Cottrell, unpublished data), and on *C. sanguinea* in England (Tavares 1979). The geographical range of each of these coccinellids overlaps with the range of *H. axyridis* in the USA (see Gordon 1985), and all three were found, albeit rarely, at the feeding site in this study. The fact that none were infected with *H. virescens* probably was due to the apparently small population size of the three species in and around the study site. Whether or not *H. virescens* can spread from *H. axyridis* to another species may depend on the frequency and duration of interspecific contact between adults. Although overwintering aggregations of mixed species (Majerus 1994a; Christian 2001) are likely to encourage interspecific contact, the commonness of physical contact between different species at feeding sites has not been reported, to my knowledge.

Although season-long infection rates were not influenced significantly by host gender in 2003 and 2004, variability in infection rates between collection dates was more pronounced for females than males, at least in 2003. Garcés and Williams (2004) found that infection of *H. axyridis* males and females in July through August 2002 was 11 and 23%, respectively, but infection of males and females in October through November 2002 was 75 and 20%, respectively; males were more abundant than females in fall collections.

The fact that infection of females was not correlated with female abundance in either season, and infection of males was correlated with male abundance only in 2003, negates the observation of any trend between infection rate and host abundance from one year to the next. Undoubtedly, the parasite must depend on the availability of hosts to sustain its population in a given locality. A number of host rather than parasite-related factors could account for the lack of consistent correlations. One such factor could be the age structure of beetles at the feeding site. The potential overlap of generations during the summer and early fall might correspond, in some instances, with the variability in infection rates. The proportion of newly-metamorphosed *H. axyridis* males might equal or exceed the proportion of older, overwintered individuals at the study site, even though *H. axyridis* are known to live up to three years (Savoïskaya 1970, in Nalepa et al. 1996). Since physical encounters between host conspecifics, rather than parasite-induced dispersal or wind dispersal, are thought to precipitate the spread of *H. virescens* ascospores throughout a host population, infection rates would typically be higher for overwintered rather than new generation adults at the feeding site. *H. axyridis* can produce 2–4 overlapping generations per year (see LaMana and Miller 1996; Katsoyannos et al. 1997; Nault and Kennedy 2003; Bazzocchi et al. 2004), depending on climate. Newly-emerged first generation *H. axyridis* adults were found on a podocarp tree (*Podocarpus* sp.), feeding on aphids, in late February 1993 in southern Mississippi (Tedders and Schaefer 1994). Unfortunately, the age (or generation) of *H. axyridis* adults collected in this study was not assessed. Individuals with a deep-red elytral color may represent older (e. g., overwintered) adults and those with a pale yellow-orange elytral color may represent new generation adults, but no attempt to monitor the frequency of these different color patterns at each collection date was made. No black morphs were ever seen at the site.

Zerm and Adis (2004) revealed that the age structure of populations of the carabid, *P. aequinoctialis bifasciata,* can influence the prevalence of its parasite, *L. phaeoxanthae* inhabiting an Amazonian floodplain in Brazil. During periods of low abundance (April to May 1998April to May 1999) a disproportionate number of beetles represented the older rather than the new generation. Thus, fully-sclerotized mature rather than immature carabid females were more often infected. Larval and pupal stages of beetle hosts are probably never infected, since several weeks are required for *H. virescens* to develop from an ascospore to a mature thallus (Strandberg and Tucker 1974; De Kesel 1993). New generation beetles would not harbor mature thalli, because developing thalli would be shed along with the exuvia at each molt. Perhaps, mating and non-mating physical contacts between *H. axyridis* adults of different generations, during summer and fall, increases the opportunities for persistence of *H. virescens* within the host population.

Although coccinellid abundance can fluctuate from week to week, due to random movement into and away from the site (Dutcher et al. 1999), the abundance of potential prey on pecan foliage may have impacted *H. axyridis* abundance at the site. Several studies have shown that the abundance of *H. axyridis* and other coccinellids can increase in relation to an abundance of aphids (Edelson and Estes 1987; LaRock and Ellington 1996; Brown 2004; Rutledge et al. 2004), leading to an influx of coccinellids from neighboring habitats. Aphid populations were not monitored in this study.

Weather conditions might have influenced the rate of infection at the study site. Although temperature and humidity were very similar at the site during the time that beetles were collected, monthly rainfall and temperature readings would probably provide a better assessment of whether or not weather was influential. The development of *H. virescens* from ascospore to mature thallus requires nearly a month (unpublished observations). Warm and humid conditions appear to promote thallus development and transmission in the laboratory (Riddick and Schaefer 2005), suggesting that these conditions would be conducive for *H. virescens* transmission to uninfected hosts in the field. Welch et al. 2001 revealed that the prevalence of *H. virescens* infection of *A. bipunctata* was more pronounced in central London than at the periphery of the city in May 1999. The slightly warmer temperatures in the center of London, in conjunction with the pollution caused by urbanization, might have provided conditions for sustained aphid population growth, leading to *A. bipunctata* reproduction and intergenerational mating (Welch et al. 2001). Urbanization and pollution from cars have been linked to aphid population growth (see Dohmen et al. 1984; Bolsinger and Flückiger 1987).

### Thallus density and distribution

The observation that *H. axyridis* males rather than females usually had more *H. virescens* on the integument suggests that male beetles are potentially more active at feeding sites. Consequently, males could contact more conspecifics; which increases the opportunities for spread of this fungus. Riddick and Schaefer (2005) observed that overwintering males harbored more *H. virescens* thalli than females. They suggested that males were more active at the site, resulting in more bodily contact between individuals and increased opportunities for spread of infection to males.

The density and distribution of thalli on female beetles in this study closely resembled what would be expected if transmission occurred when beetles mated. According to a sexual transmission hypothesis (see Welch et al. 2001), the positioning of thalli on the dorsum of females and ventrum of male conspecifics reflects the position of a male on top of a female while *in-copula.* For example, Welch et al. (2001) indicated that 75% of *A. bipunctata* males collected in June 1999 in central London had *H. virescens* thalli on the ventrum only, 16.7% had thalli on the dorsum only, and 8.3% had thalli on both the ventrum and dorsum. Some 69% of *A. bipunctata* females from the same sampling period and location had thalli on the dorsum only, 8.7% had thalli on the ventrum only, and 21.7% had thalli on both dorsum and ventrum. In addition, Garcés and Williams (2004) stated that *H. virescens* infection was concentrated on the ventroposterior of *H. axyridis* males and the dorsoposterior of females found in crop fields in northern Ohio in summer 2002.

The observation that males usually had just as many, or more, mature thalli on the dorsum as on the ventrum, as seen in the seasonal averages 2003 and 2004 in this study, was not expected in light of previous studies. The observation that approximately 67 and 96% of *H. axyridis* males and females, respectively (from the seasonal average 2004), had *H. virescens* mature thalli on the dorsum in this study corroborates with observations of thallus density and distribution on beetles found overwintering in an observation tower in a recent study (Riddick and Schaefer 2005). Clearly, a sexual transmission hypothesis cannot account for prevalence of thalli on the dorsum of male beetles, since males are thought to transfer *H. virescens* from the ventrum to the dorsum of females while mating.

One explanation for the occurrence of *H. virescens* on the dorsum of *H. axyridis* in this study is that a significant proportion of infected beetles at the feeding sites could very well represent overwintered adults. Beetles overwinter within aggregations and non-mating associated spread of *H. virescens* might occur at these sites, prior to the onset of cold weather. An alternative explanation for the prevalence of *H. virescens* thalli on the dorsum of male beetles is that males often contact conspecific males at the feeding site. Majerus (1994 a) stated that reproductively mature males often mount conspecifics, irrespective of gender, when encounters arise in the field. A contact rather than airborne pheromone may or may not be involved in attraction of males to receptive females (Hemptinne et al. 1996; Hemptinne et al. 1998). In either case, males must physically contact the integument of another individual, by using maxillary palpae (Hemptinne et al. 1998) or antennae (Jourdan et al. 1995), before species recognition can occur. Since some coccinellids mate more than once and a mating pair can remain *in-copula* for several hours (Majerus 1994b), it is reasonable to suggest that an active *H. axyridis* male will encounter numerous conspecific males and females at the feeding site each growing season. Perhaps, the brief time period of physical contact between two males is sufficient for expulsion of ascospores from mature thalli (i.e., perithecium, proper) on the ventrum of one male onto the dorsum of the other male. The conditions favorable to ascospore expulsion, in relation to body mass and time of bodily contact between paired conspecific beetles, are not well known.

In conclusion, the detection of the parasitic fungus *H. virescens* on the multicolored Asian lady beetle in Mississippi is reported here for the first time. The predominance of mature thalli on the dorsum and ventrum of *H. axyridis* males in the field in summer and early fall suggest that behaviors other than heterosexual mating contribute to the distribution of *H. virescens* on *H. axyridis.* Horizontal transmission of *H. virescens* onto *H. axyridis* adults of different generations, at feeding sites, might be vital to maintaining stable populations of the fungus from year to year.
